# Cell surface Glut1 levels distinguish human CD4 and CD8 T lymphocyte subsets with distinct effector functions

**DOI:** 10.1038/srep24129

**Published:** 2016-04-12

**Authors:** Gaspard Cretenet, Isabelle Clerc, Maria Matias, Severine Loisel, Marco Craveiro, Leal Oburoglu, Sandrina Kinet, Cédric Mongellaz, Valérie Dardalhon, Naomi Taylor

**Affiliations:** 1 Institut de Génétique Moléculaire de Montpellier, Centre National de la Recherche Scientifique UMR5535, Université de Montpellier, F-34293 Montpellier, France

## Abstract

CD4 and CD8 T lymphocyte activation requires the generation of sufficient energy to support new biosynthetic demands. Following T cell receptor (TCR) engagement, these requirements are met by an increased glycolysis, due, at least in part, to induction of the Glut1 glucose transporter. As Glut1 is upregulated on tumor cells in response to hypoxia, we assessed whether surface Glut1 levels regulate the antigen responsiveness of human T lymphocytes in both hypoxic and atmospheric oxygen conditions. Notably, Glut1 upregulation in response to TCR stimulation was significantly higher in T lymphocytes activated under hypoxic as compared to atmospheric oxygen conditions. Furthermore, TCR-stimulated human T lymphocytes sorted on the basis of Glut1-Lo and Glut1-Hi profiles maintained distinct characteristics, irrespective of the oxygen tension. While T cells activated in hypoxia divided less than those activated in atmospheric oxygen, Glut1-Hi lymphocytes exhibited increased effector phenotype acquisition, augmented proliferation, and an inverted CD4/CD8 ratio in both oxygen conditions. Moreover, Glut1-Hi T lymphocytes exhibited a significantly enhanced ability to produce IFN-γ and this secretion potential was completely dependent on continued glycolysis. Thus, Glut1 surface levels identify human T lymphocytes with distinct effector functions in both hypoxic and atmospheric oxygen tensions.

The response of a T lymphocyte to antigen stimulation is conditioned by the cell’s energetic and biosynthetic resources. T cell proliferation and effector function require the generation of ATP, phospholipids, nucleotides and NADPH. Their production is regulated by an augmented nutrient transport and utilization. Interestingly though, different T lymphocyte subsets display distinct metabolic profiles resulting from the differential utilization of glucose, fatty acids, and amino acids such as leucine and glutamine. T effector cells exhibit much higher glycolysis than suppressive regulatory T cells (Tregs) and differentiation of the latter subset is dependent on fatty acid oxidation^1^,^2^,^3^,^4^. Furthermore, the generation of T effector cells, but not regulatory T cells, requires a high level of amino acid metabolism^5^,^6^,^7^,^8^.

In the context of glucose utilization, its transport into cells is often a rate-limiting step in its metabolism. Indeed, Glut1, the major glucose transporter in T lymphocytes (reviewed in^9^), is not expressed at significant levels on the surface of quiescent T cells^10^,^11^,^12^ but is highly upregulated following TCR or cytokine stimulation^12^,^13^,^14^,^15^,^16^,^17^,^18^,^19^. Many studies have found that increasing glycolysis results in enhanced effector function, monitored as a capacity to produce IFN-γ^1^,^20^,^21^,^22^,^23^. Conversely, decreased glycolysis has been shown to inhibit both IFN-γ and IL-17 production^24^,^25^,^26^,^27^,^28^. However, a contradictory phenomenon has also been reported with T lymphocytes segregated on the basis of high glucose uptake exhibiting a terminally differentiated state with decreased effector function^21^. Notably though, the ensemble of these studies were all performed in a murine system and it is not known whether the level of glucose transport *per se*, irrespective of downstream glycolytic enzymes, modulates the fate of human T lymphocytes.

It is also important to note that T lymphocyte responsiveness can be conditioned by oxygen tension. In hypoxic conditions, there is a stabilization of the hypoxia-inducible transcription factors, HIF-1α and HIF-2α^29^. Moreover, HIF-1α enhances glycolysis and Th17 effector function but its effects on Foxp3 transcription and the associated differentiation of murine Treg differentiation are controversial^30^,^31^,^32^,^33^,^34^. Hypoxic conditions can also potentially augment Glut1 levels on T lymphocytes^35^ by interaction of HIF-1 with a consensus site in the *glut1* promoter^36^,^37^. This may have important *in vivo* significance as oxygen tensions in lymphoid tissues range from 0.5–4.5% and tumor microenvironments are often hypoxic^38^,^39^,^40^. However, whether and how Glut1 expression can predict the capacity of a human T lymphocyte to respond to antigen stimulation is unclear. Here, we report that cell surface induction of Glut1 conditions the fate of human CD4 and CD8 T lymphocytes in hypoxic as well as atmospheric oxygen conditions, with proliferation and effector function increasing with Glut1 expression.

## Results

### The glucose transporter Glut1 is highly upregulated in hypoxic conditions but TCR-induced T cell proliferation is significantly lower than at atmospheric oxygen

**Figure 1:**
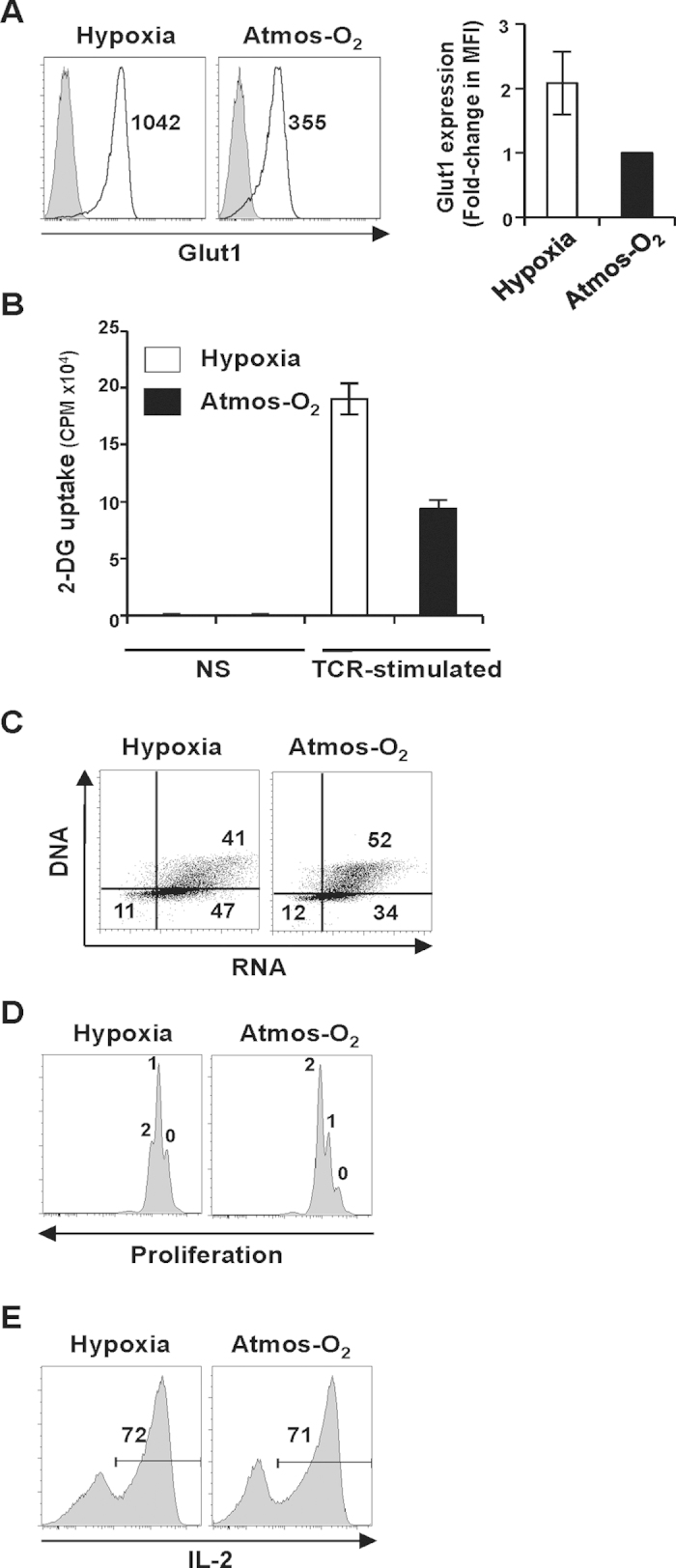
Glut1 expression and glucose uptake are significantly increased following activation of T cells under hypoxic conditions. (**A**) Human CD3^+^ T cells, activated by anti-CD3/anti-CD28 monoclonal antibodies, were cultured under hypoxic (2%) or atmospheric (Atmos-O_2_, 20%) oxygen conditions. Levels of the Glut1 glucose transporter were assessed at day 2–3 following activation and representative histograms are shown with Glut1 expression and control staining in black and filled grey histograms, respectively. Mean fluorescence intensities are indicated. The fold change in the MFI of Glut1 staining between atmospheric and hypoxic oxygen conditions was quantified in 6 independent experiments with the MFI in the former arbitrarily set at 1. Means ± SD are presented. (**B**) Glucose uptake was assessed in non-stimulated (NS) and TCR-stimulated T cell populations (1 × 10^6^) by incubation with 2-deoxy-D[1-^3^H]glucose (2-DG, 0.1 mM) for 10 min at RT. Uptake is expressed as mean counts per minute (CPM) for triplicate samples in 1 of 3 representative experiments; p < 0.05 by 2-tailed Mann-Whitney and Student’s t-test. (**C**) Cell cycle status was monitored by simultaneous staining of DNA and RNA with 7-aminoactinomycin D (7-AAD) and pyronin Y (PY), respectively. The percentages of cells in G1b (LR quadrant) and S/G2/M (UR quadrant) are indicated and are representative of 1 of 3 independent experiments. (**D**) T cell proliferation was monitored by CFSE labeling and dilution of the fluorescent dye was assessed at day 3. Each division peak is noted and represents 1 of 3 experiments. (**E**) IL-2 production was monitored at day 7 of stimulation following a 4 h PMA/ionomycin stimulation in the presence of brefeldin (**A**). Cells were fixed, permeabilized and intracellular IL-2 levels were analysed by staining with a fluorochrome-coupled antibody. Data are representative of 2 independent experiments.

As indicated above, the oxygen tensions to which lymphocytes are exposed *in vivo* are dramatically lower than the 20–21% oxygen levels present in standard incubators. Furthermore, as the tumor microenvironment is often hypoxic, lymphocytes that infiltrate into tumors can be exposed to oxygen conditions that are often <1–2%. The capacity of T cells to respond to TCR stimulation requires increased metabolism, and it is interesting to note that in tumor cells, hypoxia results in the upregulation of the Glut1 glucose transporter, potentially allowing an increased level of glycolysis^29^. We therefore assessed whether the TCR-mediated upregulation of Glut1 on primary T cells is influenced by oxygen tension. Notably, Glut1 transporter expression was significantly higher in human T cells activated under hypoxic conditions with a mean increase in Glut1 fluorescence intensity of 2-fold in 6 different donors (Fig. 1A). Moreover, this increase in Glut1 levels was associated with a significantly augmented glucose transport, monitored as a function of ^3^H-2-deoxyglucose uptake (p < 0.05; Fig. 1B). However, in hypoxic conditions, there was a lower percentage of cells in S/G_2_/M (Fig. 1C), resulting in a decreased division. While the vast majority of cells in hypoxia had undergone a single division by day 3 post stimulation, the corresponding lymphocytes at 20% O_2_ had undergone two divisions (Fig. 1D). Despite the lower proliferation (Fig. 1D), T lymphocytes in both conditions maintained the capacity to produce IL-2 (Fig. 1E).

### Ectopic Glut1 expression results in increased TCR-induced proliferation of human T lymphocytes

**Figure 2:**
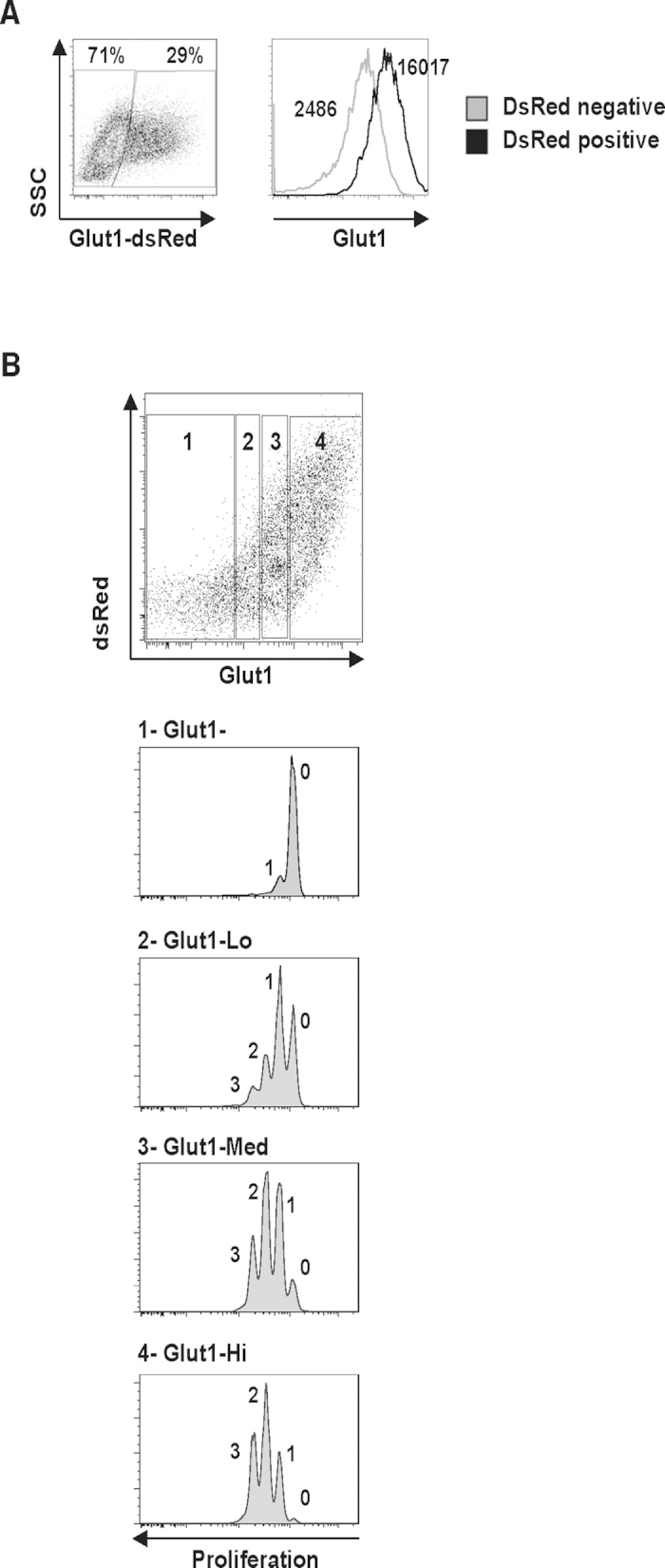
Ectopic Glut1 expression resulted in a significantly increased level of TCR-induced T cell proliferation. (**A**) CD4^+^ T cells were transduced with a dsRed-expressing Glut1 lentiviral vector. Expresssion of Glut1 in dsRed + (black lines) and dsRed- cells (grey line) are shown in a representative histogram and the respective MFIs are indicated. (**B**) To determine the relative proliferation of Glut1- versus Glut1+ and Glut1-overexpressing cells, lymphocytes were labeled with the VPD450 dye and cell division was monitored 2 days post transduction with the dsRed vector. Division profiles of cells expressing different levels of Glut1 were assessed on gated subsets, denoted as Glut1- (1), Glut1-Lo (2), Glut1-Med (3) and Glut1-Hi (4), and the number of division peaks are indicated. Data are representative of 3 independent experiments.

Given the role of Glut1 in T cell metabolism, it was intriguing that T lymphocytes activated under hypoxic conditions expressed higher levels of this transporter but underwent decreased proliferation as compared to T lymphocytes activated in atmospheric O_2_ conditions. While we hypothesized that this was due to the significantly higher amount of energy that is generated under aerobic versus anaerobic conditions, it was important to determine whether Glut1 expression *per se* was associated with a higher level of TCR-induced proliferation in human T cells. To test this hypothesis, we introduced ectopic Glut1 into mature human T cells by lentiviral transduction of a Glut1-dsRed fusion construct downstream of the SFFV promoter. This resulted in an approximate 5-fold increase in Glut1 levels as monitored by mean fluorescence intensity (Fig. 2A). We then assessed whether Glut1 levels were directly associated with changes in the proliferation profiles of T lymphocytes activated at 20% oxygen, and this was indeed the case. The majority of cells with low Glut1 (defined as population 1) had not divided whereas more than 70% of T cells expressing high levels of Glut1 (defined as population 4) had undergone 2 or more divisions (Fig. 2B). Thus, for T cells activated in atmospheric oxygen conditions, Glut1 levels directly correlate with the proliferation potential of the lymphocyte.

### TCR-induced Glut1 levels distinguish T lymphocyte subsets with distinct proliferation and CD4/CD8 profiles

**Figure 3:**
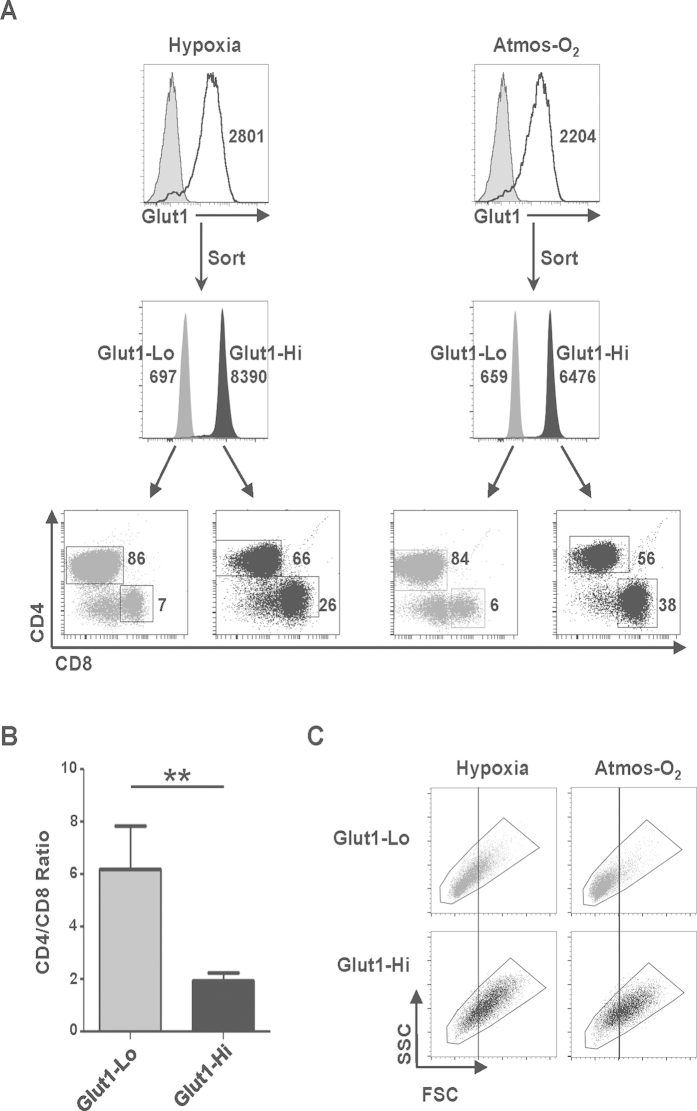
Differential Glut1 expression on TCR-activated T cells is associated with distinct repartitions of CD4 to CD8 T cells. (**A**) Purified human CD3^ + ^ T cells were VPD450-labeled and TCR-activated in hypoxic and atmospheric oxygen tensions. Two days post stimulation, T cells were stained for Glut1 expression and cells with the 5–10% lowest and highest levels of surface Glut1 were sorted on a FACSAria cell sorter as shown and are hereby designated as Glut1-Lo and Glut1-Hi, respectively. The repartition of CD4 and CD8 T cells within each subset was assessed immediately after sorting and representative dot plots are shown. (**B**) Quantification of the ratio of CD4/CD8 T cells in the sorted Glut1-Lo and Glut1-Hi subsets. Mean ratios ± SEM of 5 independent experiments are presented and statistical differences were determined by a 2-tailed Mann-Whitney test; **p < 0.01. (**C**) The forward (FSC) and side (SSC) scatter profiles of the Glut1-Lo and Glut1-Hi FACS-sorted subsets activated at hypoxia and atmospheric oxygen are presented. Data are representative of 3 independent experiments.

Based on the results described above, it was of interest to determine whether endogenous Glut1 expression influences T cell fate under hypoxic and/or atmospheric oxygen conditions. To this end, T lymphocytes were activated in both conditions and 48 h post TCR stimulation, lymphocytes were isolated as a function of surface Glut1 expression. Specifically, the 10% of lymphocytes expressing the lowest and highest levels of Glut1 were isolated on a FACSAria cell sorter and are hereafter referred to as Glut1-Lo and Glut1-Hi, respectively (Fig. 3A). Interestingly, at both 1% and 20% oxygen conditions, the percentages of CD8 T cells in the Glut1-Hi subsets were significantly higher than in the Glut1-Lo subsets, with a mean CD4/CD8 ratio of 6 in the Glut1-Lo subset and 3 in the Glut1-Hi subset (p < 0.01, Fig. 3A,B). It is important to note that this was not due to differences in the percentages of dividing versus non-dividing cells as proliferation at 48 h was minimal (Supplementary Figure 1). Furthermore, Glut1-Lo and Glut1-Hi subsets did not appear to be differentially polarized as the relative mRNA levels of transcription factors such as Tbet and GATA3 and effector cytokines such as IFNγ and IL-17 were not altered in the two subsets (data not shown). Consistent with a higher metabolic activity in Glut1-Hi cells, their forward/side scatters were significantly higher than those of Glut1-Lo cells, both at 1% and 20% oxygen tensions (Fig. 3C). Notably, the sorted Glut1-Lo and Glut1-Hi T cells remained distinct with significantly higher levels of Glut1 detected on the latter 24 h post FACS isolation, in both hypoxic and atmospheric oxygen conditions (data not shown).

**Figure 4:**
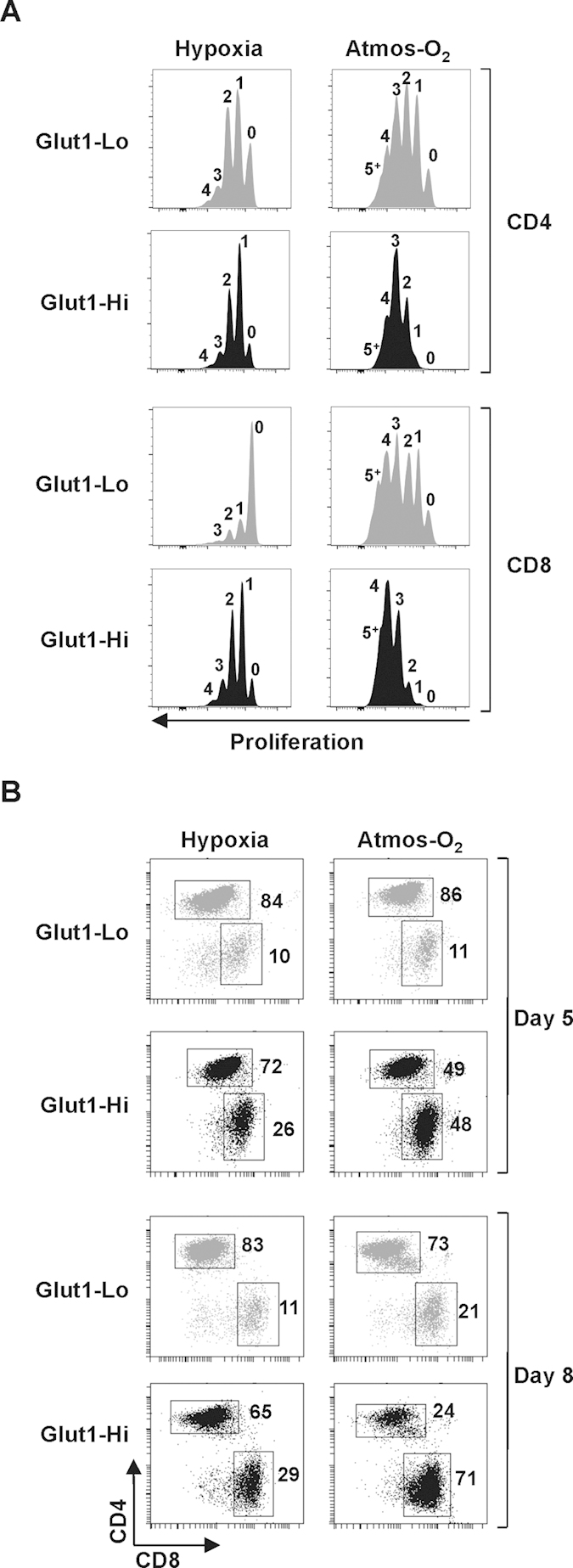
Glut1-Hi T cells maintain a distinct phenotype with enhanced proliferation and an increased proportion of CD8+ lymphocytes. (**A**) T lymphocytes activated in hypoxia and atmospheric oxygen were sorted at day 2 on the basis of Glut1 expression. The proliferation of Glut1-Lo and Glut1-Hi subsets were monitored at day 5 post stimulation as a function of VPD450 fluorescence and representative histograms showing the number of divisions are presented. (**B**) The repartition between CD4 and CD8 T cells within the Glut1-Lo and Glut1-Hi subsets are presented at days 5 and 8 post stimulation. The percentages of cells within each quadrant are indicated. Data are representative of 3 independent experiments.

The fate of Glut1-Lo and Glut1-Hi cells was maintained during continued culture of the cells in the presence of IL-2. While all Glut1 subsets proliferated to a greater extent at 20% than 1% oxygen, in agreement with the data presented in Fig. 2, a greater percentage of Glut1-Hi cells divided as compared to Glut1-Lo cells (Fig. 4A). These data corresponded to a striking bias towards CD8 T cells within the Glut1-Hi subset at 20% oxygen; more than 70% of lymphocytes in the Glut1-Hi subset were CD8+ by day 8 as compared to 21% in the Glut1-Lo subset (Fig. 4B). At 1% oxygen, the percentage of CD8 T cells in the Glut1-Hi subset was also augmented relative to the Glut1-Lo subset but the differences were less marked; 29% in Glut1-Hi compared to 11% in the Glut1-Lo subset (Fig. 4B). This correlated with a decreased proliferation of all subsets at 1% oxygen relative to 20% oxygen. The ensemble of these data demonstrates the importance of the initial TCR-induced cell surface upregulation of Glut1 in modulating the subsequent fate of these lymphocytes. T cells selected on the basis of high Glut1 expression exhibit a significantly higher proliferation rate than their Glut1-Lo counterpart and this distinction results in a selective advantage for the CD8 T cell population.

### Distinct phenotypes and proliferation profiles of Glut1-Lo and Glut1-Hi T cells

**Figure 5:**
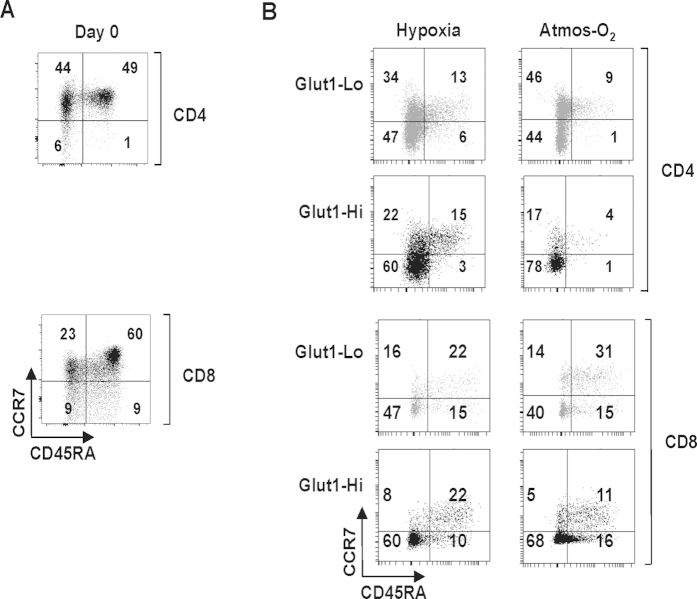
T lymphocytes expressing high surface Glut1 levels exhibit increased acquisition of an effector phenotype. (**A**) The naïve, memory and effector phenotypes of freshly isolated CD4 and CD8 T lymphocytes were monitored as a function of CCR7 and CD45RA expression (CCR7+CD45RA+, naïve; CCR7+CD45RA-, central memory; CCR7-CD45RA-, effector memory). Representative plots are shown. (**B**) T lymphocytes were TCR-activated for 2 days and sorted on the basis of Glut1-Lo and Glut1-Hi expression. Purified subsets were then maintained in culture in the presence of rIL-2 in hypoxia and atmospheric oxygen as indicated. The naïve, memory and effector phenotypes of the CD4 and CD8 populations were distinguished by CCR7/CD45RA staining. Representative plots of 3 independent experiments are presented and the percentages of T cells in each quadrant are indicated.

To follow the phenotype of FACS-sorted Glut1-Lo and Glut1-Hi T cells, we assessed CCR7 and CD45RA expression. Naïve T cells are identified as CCR7+/CD45RA+ while central memory cells are CCR7+/CD45RA- and effector memory cells are CCR7-/CD45RA-. In freshly isolated quiescent T lymphocytes from healthy adult donors, approximately 50% of cells are naïve (Fig. 5A). Upon TCR engagement, naïve cells rapidly lose CD45RA expression, with the vast majority of cells acquiring memory or effector phenotypes. Interestingly, acquisition of central memory and effector memory phenotypes was similar in 1% and 20% oxygen conditions, but Glut1 levels significantly influenced T cell fate. The percentage of effector memory T cells, characterized as CCR7-CD45RA-, was significantly higher in the Glut1-Hi than the Glut1-Lo subsets, with percentages ranging from 60–78% versus 40–47%, respectively (Fig. 5B). These data show that acquisition of an effector memory phenotype correlates with surface Glut1 levels.

**Figure 6:**
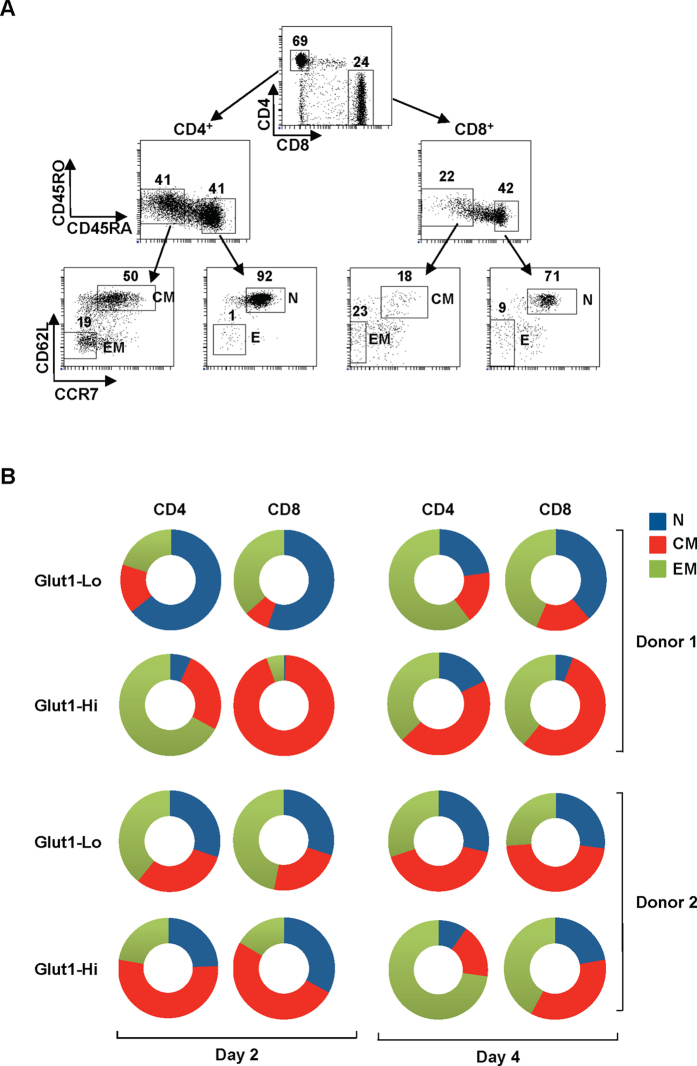
TCR-induced Glut1 expression varies as a function of T cell phenotype and the kinetics of T cell activation. (**A**) Sorting strategy for isolation of freshly isolated CD4 and CD8 T cell subsets. Representative plots show CD4/CD8, CD45RO/CD45RA, and CD62L/CCR7 repartitions. Naïve (N), central memory (CM) and effector memory (EM) CD4 and CD8 subsets were sorted as CD45RA+CD45RO-CD62L+CCR7+, CD45RO+CD45RA-CD62L+CCR7+, and CD45RO+CD45RA-CD62L-CCR7- phenotypes, respectively. Percentages of cells in each gate are indicated and subsets were sorted on a FACSAria to >90% purity. (**B**) Sorted subsets were activated and Glut1 expression was assessed at days 2 and 4 post TCR stimulation. Glut1-Lo and Glut1-Hi cells were defined as a function of the 10% lowest and highest levels of surface Glut1. Pie charts present the relative repartition of naïve, central memory and effector memory CD4 and CD8 cells within the Glut1-Lo and Glut1-Hi gates in 2 healthy donors at days 2 and 4 post TCR stimulation. Raw data for one donor are presented in Supplementary Figure 1.

Nevertheless, based on the experiments presented above, we could not exclude the possibility that the differences detected in T cells expressing distinct levels of Glut1 levels were determined by the cell’s initial phenotype. Indeed, CD4 and CD8 T cells are heterogeneous with naïve (N), central memory (CM) and effector memory (EM) subsets presenting lymphocytes with diverse response histories. We therefore FACS-sorted these six subsets (CD4-N, CD8-N, CD4-CM, CD8-CM, CD4-EM, CD8-EM) from fresh peripheral blood samples (Fig. 6A) and assessed whether they exhibited differences in the kinetics of TCR-induced Glut1 upregulation and proliferation. TCR stimulation resulted in a rapid Glut1 upregulation in all T cell subsets. However, as shown in Fig. 6B, there is clearly diversity between donors; while there was a higher percentage of naïve CD4 and CD8 T cells within the Glut1-Lo population at day 2 post stimulation, a significant percentage of naïve CD4 and CD8 T cells from donor 2 were also Glut1-Hi. A higher percentage of CM cells were present in the Glut1-Hi than the Glut1-Lo subsets for both CD4 and CD8 T cells. Importantly, the percentages of N, CM and EM T cells within the Glut1-Lo and Glut1-Hi subsets changed with time post stimulation. At day 4 post stimulation, varying percentages of N, CM, and EM populations were found in both Glut1-Lo and Glut1-Hi subsets (Fig. 6B and Supplementary Figure 1). While N, CM and EM T cells all underwent extensive TCR-induced proliferation by day 4, cell surface Glut1 levels on all subsets were higher on T cells that had undergone at least 1 round of division. Moreover, the surface expression of Glut1 on T cells following TCR stimulation is not static but rather evolves with time (Supplementary Figure 1). These data are in agreement with previous studies showing that surface Glut1 decreases to undetectable levels as T lymphocytes return to a resting state^12^.

### Glut1-Hi cells exhibit enhanced effector function

**Figure 7:**
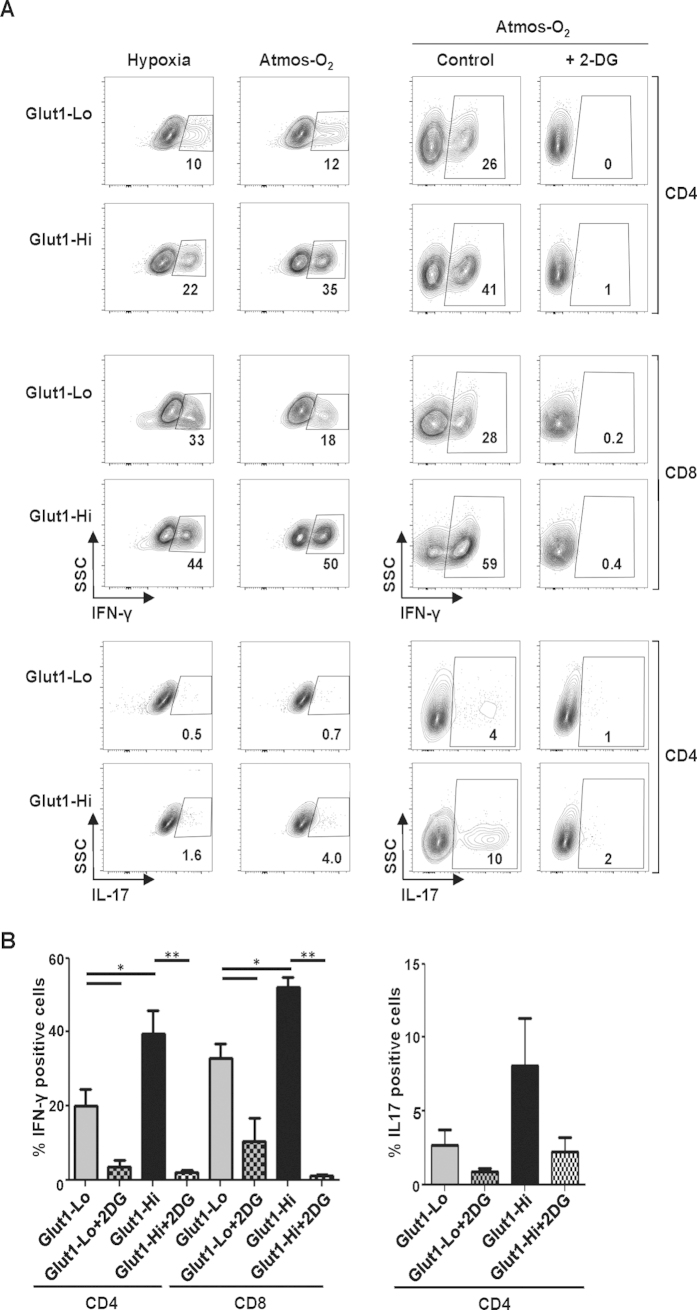
Surface Glut1 levels modulate the cytokine secretion potential of CD4 and CD8 T lymphocytes at both hypoxic and atmospheric oxygen conditions. (**A**) TCR-stimulated CD3^+^ human T cells were FACS-sorted at day 2 on the basis of Glut1 levels and cultured for an additional 3 days at either hypoxia or atmospheric oxygen as indicated. Production of IFN-γ by CD4 and CD8 T cells as well as IL-17 by CD4 T cells was monitored in the Glut1-Lo and Glut1-Hi subsets. Intracellular staining was performed 4 h post PMA/ionomycin treatment in the presence of brefeldin A (left plots). IFN-γ and IL-17 production was also monitored simultaneously in CD4 and CD8 subsets that were restimulated with PMA/ionomycin in the absence or presence of 2-deoxyglucose (2-DG) and data from 1 of 5 independent experiments are shown (right plots). (**B**) Quantification of IFN-γ and IL-17 production in Glut1-Hi and Glut1-Lo subsets as a function of restimulation in the absence or presence of 2-DG. Means ± SEM of 5 independent experiments are presented and statistical difference was monitored by a 2-tailed Mann-Whitney test; *p < 0.05; **p < 0.01.

The data presented above suggested that the effector function of human T cells might be more affected by surface Glut1 expression than by oxygen concentration. Notably, IFN-γ secretion was significantly higher in the Glut1-Hi subsets of both CD4 and CD8 T cells than in the Glut1-Lo counterparts; in CD4 T cells, the mean percentage of IFN-γ-expressing cells increased from 20% in the Glut1-Lo subset to 39% in the Glut1-Hi subset while in CD8 T cells, it increased from 32% to 53% (p < 0.05; Fig. 7A,B). The same trend was also detected for IL-17 secretion by CD4 T cells, increasing from a mean of 3% to 8% but this difference was not significant due to variability between donors (Fig. 7A,B). Based on these data, we assessed whether the relative contribution of glycolysis to cytokine secretion differed in Glut1-Lo versus Glut1-Hi T cells. In this regard, previous studies have elegantly shown that inhibiting glycolysis in murine T cells results in a significant attenuation of IFN-γ production while conversely, increasing glycolysis promotes IFN-γ secretion from murine T cells^20^,^22^,^23^,^25^,^41^,^42^. Indeed, we found that blocking glycolysis in human T cells with a non-metabolizable 2-deoxyglucose (2-DG) analogue dramatically inhibited IFN-γ secretion. Importantly, this inhibition occurred in both Glut1-Lo and Glut1-Hi subsets (p < 0.05; Fig. 7B). Thus, maintaining glucose metabolism is critical for the IFN-γ secretion potential of T lymphocytes, irrespective of their level of Glut1 expression. However, under conditions where glycolysis is maintained, the potential of T cells to secrete IFN-γ is significantly enhanced in lymphocytes expressing high Glut1 levels. These data attest to the importance of nutrient resources and their intracellular transport in modulating the function of human T lymphocytes.

## Discussion

Recent findings reveal the critical role of metabolic programs in conditioning T cell activation and differentiation, suggesting new strategic avenues for modulating T cell function. As many studies have found that genetic manipulations resulting in enhanced glycolysis lead to augmented T cell effector function^1^,^20^,^21^,^22^,^23^,^26^,^43^, it was of interest to determine whether differences in Glut1 surface expression are associated with distinct cell fates. Glut1 is rapidly upregulated following T cell stimulation, peaking at approximately 2–3 days post TCR engagement and then slowly returning to baseline levels^12^,^15^,^16^,^18^,^19^. Here we show that Glut1 levels distinguish the behavior of human T lymphocytes following TCR stimulation. Notably, segregating T lymphocytes on the basis of Glut1 levels at an early time following TCR stimulation (2 days), identified subsets with unique phenotypic and functional traits. Glut1-Hi CD4 as well as CD8 T lymphocytes preferentially acquired an effector phenotype with enhanced effector function.

Glut1 regulation is complex, controlled at the level of transcription, translation and transport to the cell surface. This regulation is critical as glucose uptake across the plasma membrane is often the rate-limiting step in the production of ATP. In accord with the importance of Glut1 regulation, augmented Glut1 levels at the cell surface optimize the “fitness” of tumor cells to their hypoxic environment (reviewed in^44^). Our finding that hypoxia also increases TCR-induced Glut1 levels on T lymphocytes suggests a potential competition between tumor cells and infiltrating T lymphocytes for glucose. Indeed, two recent studies have elegantly shown that increased tumor glycolysis severely impedes the capacity of infiltrating T cells to control tumor growth. However, manipulations that increase T cell glycolysis within the tumor microenvironment resulted in bolstered effector functions, promoting IFN-γ secretion^22^,^23^. Furthermore, it appears that even the beneficial effects of checkpoint inhibitors (αCTLA-4, αPD-1 and αPD-L1) are regulated by T cell metabolism; glucose concentrations within the tumor increase, resulting in a higher glycolysis in infiltrating T cells^22^. While we found that human T lymphocytes divided less robustly in hypoxia, augmented cell surface levels of Glut1 may have evolved in order to promote an efficient and rapid adaptive immune response against infectious agents and tumor antigens, even in oxygen-deprived regions of the body. Indeed, the potential of CD4 as well as CD8 T lymphocytes to secrete IFN-γ is significantly enhanced in the Glut1-Hi populations.

Conversely, manipulations that decrease the glycolysis of T lymphocytes may improve the outcome of patients with T cell-mediated autoimmune disease. In this regard, it is significant that blocking glucose metabolism in murine T cells results in a significant attenuation of IFN-γ secretion^20^,^25^,^41^. In accord with these data, we found that glucose metabolism is a *sine qua non* for IFN-γ generation by activated human T cells, at least *ex vivo*. Moreover, a combined block of glycolysis and hexokinase activity reverses the development of an autoimmune disease, systemic lupus erythematosus, in a murine model^27^,^28^. As Glut1 is the major glucose transporter on T lymphocytes, these data suggest that Glut1 levels may remain elevated in autoimmune conditions. Furthermore, it is likely that the subset of T lymphocytes with high Glut1 levels early following TCR stimulation is distinct from the subset with high Glut1 levels at late time points, during the contraction phase of an immune response. This phenomenon may explain the apparent contradictions between studies reporting enhanced versus decreased effector functions for highly glycolytic murine T cells^1^,^20^,^21^,^22^,^23^. Indeed, we show here that while there is an initial enrichment of naïve T lymphocytes in the Glut1-Lo population and central memory CD8 lymphocytes in the Glut1-Hi population, surface Glut1 expression on the different subsets evolves with time post TCR stimulation. Nonetheless, it is important to note that under physiological conditions, surface Glut1 levels return to low baseline on all T cell subsets as they return to a resting state, at late time points following TCR stimulation.

In conclusion, our data reveal a robust proliferation and cytokine secretion in human T lymphocytes exhibiting an early TCR-induced upregulation of Glut1. Furthermore, cell surface expression of Glut1 on naïve, central memory and effector memory T cells can be used as a biomarker of recent T cell activation. Future studies will allow us to determine whether there is a differential proportion of Glut1-Lo and Glut1-Hi T lymphocyte subsets in immunopathological conditions such as autoimmunity, chronic inflammation and cancer, potentially opening new avenues for targeting immune responses.

## Methods

### T cell isolation and culture

CD3^+^ T cells were isolated from healthy adult donors. All experiments using primary human cells were conducted in accordance with the Declaration of Helsinki and IRB approval to the French Blood Bank (Etablissement Français du Sang). T lymphocytes were purified by negative-selection using Rosette tetramers (StemCell Technologies) and the purity was monitored by flow cytometry. Lymphocytes were cultured in RPMI medium 1640 + GlutaMAX (Gibco-Life technologies) supplemented with 10% FCS and 1% penicillin/streptomycin (Gibco-Life technologies).

T cell activations were performed using plate-bound anti-CD3 (clone OKT3, Biolegend) and anti-CD28 (clone 9.3, kindly provided by Carl June) mAbs at a concentration of 1 μg/ml. rIL-2 (100 U/ml) was added every other day starting at day 2 post-activation. Cells were maintained in a standard tissue culture incubator containing atmospheric (20%) O_2_ or an air-controlled incubator where O_2_ conditions were maintained at 1–2% by nitrogen injection (Heraeus incubator; Sanyo) and 5% CO_2_.

### Flow cytometry analyses and cell sorting

Immunophenotyping of cells was performed using fluorochrome-conjugated mAbs (BD Bioscience or eBioscience) against CD4, CD8, CD45RA, CD45RO, CCR7, CD62L and isotype controls (Beckman Coulter). Cell cycle analysis was performed by staining cells for DNA and RNA content with 7-amino-actinomycin-D (7AAD; 20 μM, Sigma) and pyronin Y (PY; 5 μM, Sigma), respectively. Proliferation was monitored by labeling cells with either CFSE (Life Technologies; 2.5 μM) or VPD450 (BD Biosciences; 1 μM) for 3 min at RT. Surface Glut1 expression was monitored by binding to the Glut1 ligand fused to eGFP as previously described^19^,^45^,^46^ (Metafora biosystems). For sorting of cells based on Glut1 expression, 60 × 10e^6^ cells were stained at day 2 post stimulation and the 10% of lymphocytes expressing the highest and lowest levels of Glut1 were sorted on a FACSAria (BD Biosciences). For sorting of CD4 and CD8 naïve, central memory and effector memory subsets, freshly isolated T cells were stained with fluorochrome-conjugated mAbs against CD4, CD8, CD45 RA, CD45RO, CCR7 and CD62L. Cells were FACS-sorted based on the gating strategy shown in Fig. 6. For intracellular cytokine staining, lymphocytes were re-activated with PMA (Sigma-Aldrich; 100 ng/ml) and ionomycin (Sigma-Aldrich; 1 μg/ml) in the presence of brefeldin A (Sigma-Aldrich; 10 ug/ml) for 3.5–4h at 37 °C. Re-activations were performed either in the absence or presence of 2-deoxyglucose (50 mM, Sigma) to inhibit glycolysis. Intracellular staining for IL-2, IFN-γ and IL-17 was performed following fixation/permeabilization using the eBioscience kit or using 4% PFA (Thermo-scientific)/ 0.1% saponin (Sigma). Cells were analysed on a CantoII or LSRII-Fortessa (BD Biosciences) and data were analysed using Diva (BD Biosciences) or FlowJo (Tree Star) software.

### Virus production and T cell transduction

For expression of ectopic Glut1, a Glut1-dsRed fusion construct^45^ was inserted into the HIV1-derived pCSGW lentiviral vector, harboring the SFFV promoter. Self-inactivating single-round HIV-1 virions were generated by CaCl_2_ transfection of 293T cells with pCSGW-Glut1-dsRed together with the 8.91 Gag-Pol packaging construct and the CMV-VSV-G plasmid. Viral supernatant was harvested 48 h post transfection and concentrated by ultracentrifugation (25,000 rpm) for 2 h at 4 °C. For T cell transductions, 1 × 10^6^ lymphocytes were activated for 24 h and transduced with VSV-G–pseudotyped HIV-1 vector at MOI of 1 in IL-2-containing RPMI media for 48 h. Transduction was determined as the percentage of DsRed+ cells on an LSRII Fortessa (BD Biosciences).

### Glucose uptake assays

Prior to transport analyses, T cells (0.5 × 10^6^) were starved in glucose-free RPMI for 30 min at 37 °C. Glucose uptake was initiated by addition of 2-deoxy-D[1-^3^H]glucose (2 μCi; Perkin Elmer) for 10 min at room temperature. Cells were then washed and lysed in 0.1% SDS (500 μl) and radioactivity was measured by liquid scintillation. Uptake for each cell subset is expressed as mean counts per minute (CPM) for triplicate samples and error bars indicate standard deviation (SD).

### Statistical analyses

p values were determined using a Mann-Whitney test with a 2-tailed distribution (Graph Pad Software, La Jolla, CA).

## Additional Information

**How to cite this article**: Cretenet, G. *et al.* Cell surface Glut1 levels distinguish human CD4 and CD8 T lymphocyte subsets with distinct effector functions. *Sci. Rep.* **6**, 24129; doi: 10.1038/srep24129 (2016).

## Supplementary Material

Supplementary Information

## References

[b1] Michalek R. D. *et al.* Cutting edge: Distinct glycolytic and lipid oxidative metabolic programs are essential for effector and regulatory CD4 + T cell subsets. J Immunol 186, 3299–3303, 10.4049/jimmunol.1003613 (2011). 21317389 PMC3198034

[b2] Shi L. Z. *et al.* HIF1{alpha}-dependent glycolytic pathway orchestrates a metabolic checkpoint for the differentiation of TH17 and Treg cells. J Exp Med 208, 1367–1376, 10.1084/jem.20110278 (2011). 21708926 PMC3135370

[b3] Wang R. *et al.* The transcription factor Myc controls metabolic reprogramming upon T lymphocyte activation. Immunity 35, 871–882, 10.1016/j.immuni.2011.09.021 (2011). 22195744 PMC3248798

[b4] Berod L. *et al.* De novo fatty acid synthesis controls the fate between regulatory T and T helper 17 cells. Nature medicine 20, 1327–1333, 10.1038/nm.3704 (2014). 25282359

[b5] Sinclair L. V. *et al.* Control of amino-acid transport by antigen receptors coordinates the metabolic reprogramming essential for T cell differentiation. Nat Immunol 14, 500–508, 10.1038/ni.2556 (2013). 23525088 PMC3672957

[b6] Nakaya M. *et al.* Inflammatory T Cell Responses Rely on Amino Acid Transporter ASCT2 Facilitation of Glutamine Uptake and mTORC1 Kinase Activation. Immunity 40, 692–705, 10.1016/j.immuni.2014.04.007 (2014). 24792914 PMC4074507

[b7] Macintyre A. N. *et al.* The Glucose Transporter Glut1 Is Selectively Essential for CD4 T Cell Activation and Effector Function. Cell metabolism 20, 61–72, 10.1016/j.cmet.2014.05.004 (2014). 24930970 PMC4079750

[b8] Klysz D. *et al.* Glutamine-dependent alpha-ketoglutarate production regulates the balance between T helper 1 cell and regulatory T cell generation. Science signaling 8, ra97, 10.1126/scisignal.aab2610 (2015). 26420908

[b9] Hruz P. W. & Mueckler M. M. Structural analysis of the GLUT1 facilitative glucose transporter (review). Mol Membr Biol 18, 183–193 (2001). 11681785 10.1080/09687680110072140

[b10] Rathmell J. C., Elstrom R. L., Cinalli R. M. & Thompson C. B. Activated Akt promotes increased resting T cell size, CD28-independent T cell growth, and development of autoimmunity and lymphoma. Eur J Immunol 33, 2223–2232 (2003). 12884297 10.1002/eji.200324048

[b11] Frauwirth K. A. *et al.* The CD28 signaling pathway regulates glucose metabolism. Immunity 16, 769–777 (2002). 12121659 10.1016/s1074-7613(02)00323-0

[b12] Manel N. *et al.* The HTLV receptor is an early T-cell activation marker whose expression requires de novo protein synthesis. Blood 101, 1913–1918 (2003). 12393496 10.1182/blood-2002-09-2681

[b13] Yu Q., Erman B., Bhandoola A., Sharrow S. O. & Singer A. In vitro evidence that cytokine receptor signals are required for differentiation of double positive thymocytes into functionally mature CD8(+) T cells. J Exp Med 197, 475–487 (2003). 12591905 10.1084/jem.20021765 PMC2193862

[b14] Barata J. T. *et al.* Activation of PI3K is indispensable for interleukin 7-mediated viability, proliferation, glucose use, and growth of T cell acute lymphoblastic leukemia cells. J Exp Med 200, 659–669 (2004). 15353558 10.1084/jem.20040789 PMC2212738

[b15] Jacobs S. R., Michalek R. D. & Rathmell J. C. IL-7 is essential for homeostatic control of T cell metabolism *in vivo*. J Immunol 184, 3461–3469, 10.4049/jimmunol.0902593 (2010). 20194717 PMC2980949

[b16] Wofford J. A., Wieman H. L., Jacobs S. R., Zhao Y. & Rathmell J. C. IL-7 promotes Glut1 trafficking and glucose uptake via STAT5-mediated activation of Akt to support T-cell survival. Blood 111, 2101–2111, 10.1182/blood-2007-06-096297 (2008). 18042802 PMC2234050

[b17] Swainson L. *et al.* IL-7-induced proliferation of recent thymic emigrants requires activation of the PI3K pathway. Blood 109, 1034–1042 (2007). 17023582 10.1182/blood-2006-06-027912

[b18] Chakrabarti R., Jung C. Y., Lee T. P., Liu H. & Mookerjee B. K. Changes in glucose transport and transporter isoforms during the activation of human peripheral blood lymphocytes by phytohemagglutinin. J Immunol 152, 2660–2668 (1994). 8144874

[b19] Kinet S. *et al.* Isolated receptor binding domains of HTLV-1 and HTLV-2 envelopes bind Glut-1 on activated CD4+ and CD8+ T cells. Retrovirology 4, 31 (2007). 17504522 10.1186/1742-4690-4-31 PMC1876471

[b20] Chang C. H. *et al.* Posttranscriptional control of T cell effector function by aerobic glycolysis. Cell 153, 1239–1251, 10.1016/j.cell.2013.05.016 (2013). 23746840 PMC3804311

[b21] Sukumar M. *et al.* Inhibiting glycolytic metabolism enhances CD8+ T cell memory and antitumor function. J Clin Invest 123, 4479–4488, 10.1172/JCI69589 (2013). 24091329 PMC3784544

[b22] Chang C. H. *et al.* Metabolic Competition in the Tumor Microenvironment Is a Driver of Cancer Progression. Cell 162, 1229–1241, 10.1016/j.cell.2015.08.016 (2015). 26321679 PMC4864363

[b23] Ho P. C. *et al.* Phosphoenolpyruvate Is a Metabolic Checkpoint of Anti-tumor T Cell Responses. Cell 162, 1217–1228, 10.1016/j.cell.2015.08.012 (2015). 26321681 PMC4567953

[b24] Cham C. M., Driessens G., O’Keefe J. P. & Gajewski T. F. Glucose deprivation inhibits multiple key gene expression events and effector functions in CD8+ T cells. Eur J Immunol 38, 2438–2450, 10.1002/eji.200838289 (2008). 18792400 PMC3008428

[b25] Cham C. M. & Gajewski T. F. Glucose availability regulates IFN-gamma production and p70S6 kinase activation in CD8+ effector T cells. J Immunol 174, 4670–4677 (2005). 15814691 10.4049/jimmunol.174.8.4670

[b26] Blagih J. *et al.* The energy sensor AMPK regulates T cell metabolic adaptation and effector responses *in vivo*. Immunity 42, 41–54, 10.1016/j.immuni.2014.12.030 (2015). 25607458

[b27] Yin Y. *et al.* Normalization of CD4+ T cell metabolism reverses lupus. Science translational medicine 7, 274ra218, 10.1126/scitranslmed.aaa0835 (2015). PMC5292723 25673763

[b28] Yin Y. *et al.* Glucose Oxidation Is Critical for CD4+ T Cell Activation in a Mouse Model of Systemic Lupus Erythematosus. J Immunol, 10.4049/jimmunol.1501537 (2015). PMC4684991 26608911

[b29] Semenza G. L. Oxygen homeostasis. Wiley Interdiscip Rev Syst Biol Med 2, 336–361, 10.1002/wsbm.69 (2010). 20836033

[b30] Ben-Shoshan J., Maysel-Auslender S., Mor A., Keren G. & George J. Hypoxia controls CD4+ CD25+ regulatory T-cell homeostasis via hypoxia-inducible factor-1alpha. Eur J Immunol 38, 2412–2418, 10.1002/eji.200838318 (2008). 18792019

[b31] Dang E. V. *et al.* Control of TH17/Treg Balance by Hypoxia-Inducible Factor 1. Cell 146, 772–784, 10.1016/j.cell.2011.07.033 (2011). 21871655 PMC3387678

[b32] Shi L. Z. *et al.* HIF1alpha-dependent glycolytic pathway orchestrates a metabolic checkpoint for the differentiation of TH17 and Treg cells. J Exp Med 208, 1367–1376, 10.1084/jem.20110278 (2011). 21708926 PMC3135370

[b33] Clambey E. T. *et al.* Hypoxia-inducible factor-1 alpha-dependent induction of FoxP3 drives regulatory T-cell abundance and function during inflammatory hypoxia of the mucosa. Proc Natl Acad Sci USA 109, E2784–2793, 10.1073/pnas.1202366109 (2012). 22988108 PMC3478644

[b34] Finlay D. K. *et al.* PDK1 regulation of mTOR and hypoxia-inducible factor 1 integrate metabolism and migration of CD8+ T cells. J Exp Med 209, 2441–2453, 10.1084/jem.20112607 (2011). PMC3526360 23183047

[b35] Loisel-Meyer S. *et al.* Glut1-mediated glucose transport regulates HIV infection. Proc Natl Acad Sci USA 109, 2549–2554, 10.1073/pnas.1121427109 (2012). 22308487 PMC3289356

[b36] Murakami T. *et al.* Identification of two enhancer elements in the gene encoding the type 1 glucose transporter from the mouse which are responsive to serum, growth factor, and oncogenes. J Biol Chem 267, 9300–9306 (1992). 1339457

[b37] Ebert B. L., Firth J. D. & Ratcliffe P. J. Hypoxia and mitochondrial inhibitors regulate expression of glucose transporter-1 via distinct Cis-acting sequences. J Biol Chem 270, 29083–29089 (1995). 7493931 10.1074/jbc.270.49.29083

[b38] Caldwell C. C. *et al.* Differential effects of physiologically relevant hypoxic conditions on T lymphocyte development and effector functions. J Immunol 167, 6140–6149 (2001). 11714773 10.4049/jimmunol.167.11.6140

[b39] Westermann J. & Pabst R. Distribution of lymphocyte subsets and natural killer cells in the human body. Clin Investig 70, 539–544 (1992). 10.1007/BF00184787 1392422

[b40] Sopper S. *et al.* Impact of simian immunodeficiency virus (SIV) infection on lymphocyte numbers and T-cell turnover in different organs of rhesus monkeys. Blood 10, 10 (2002). 10.1182/blood-2002-06-1644 12393472

[b41] Zheng Y., Delgoffe G. M., Meyer C. F., Chan W. & Powell J. D. Anergic T cells are metabolically anergic. J Immunol 183, 6095–6101, 10.4049/jimmunol.0803510 (2009). 19841171 PMC2884282

[b42] Cao Y., Rathmell J. C. & Macintyre A. N. Metabolic reprogramming towards aerobic glycolysis correlates with greater proliferative ability and resistance to metabolic inhibition in CD8 versus CD4 T cells. PLoS One 9, e104104, 10.1371/journal.pone.0104104 (2014). 25090630 PMC4121309

[b43] Gerriets V. A. *et al.* Metabolic programming and PDHK1 control CD4+ T cell subsets and inflammation. J Clin Invest 125, 194–207, 10.1172/JCI76012 (2015). 25437876 PMC4382238

[b44] Macheda M. L., Rogers S. & Best J. D. Molecular and cellular regulation of glucose transporter (GLUT) proteins in cancer. J Cell Physiol 202, 654–662 (2005). 15389572 10.1002/jcp.20166

[b45] Manel N. *et al.* The ubiquitous glucose transporter GLUT-1 is a receptor for HTLV. Cell 115, 449–459 (2003). 14622599 10.1016/s0092-8674(03)00881-x

[b46] Kim F. J. *et al.* HTLV-1 and -2 envelope SU subdomains and critical determinants in receptor binding. Retrovirology 1, 41, 10.1186/1742-4690-1-41 (2004). 15575958 PMC539286

