# Supplementary material for: Cell surface Glut1 levels distinguish human CD4 and CD8 T lymphocyte subsets with distinct effector functions
Source: Sci Rep. 2016 Apr 12;6:24129. doi: 10.1038/srep24129 (PMC4828702; doi:10.1038/srep24129)
Supplement: Supplementary Information [file srep24129-s1.docx]

**Cell surface Glut1 levels distinguish human CD4 and CD8 T lymphocyte subsets with distinct effector functions**

Gaspard Cretenet, Isabelle Clerc^*^, Maria Matias^*^, Severine Loisel^*^, Marco Craveiro, Leal Oburoglu, Sandrina Kinet, Cédric Mongellaz, Valérie Dardalhon^#^ and Naomi Taylor^#^

Supplementary Figure 1. **TCR-induced Glut1 expression and proliferation as a function of T cell phenotype.**

Freshly isolated CD4 and CD8 T cells were sorted on the basis of naïve, central memory and effector memory phenotypes as shown in Figure 6. Cells were labeled with VPD450 to monitor proliferation following TCR stimulation. Expression of surface Glut1 as a function of proliferation (VPD450) is shown for each subset at days 2 and 4 of stimulation. The percentages of each subset designated as Glut1-Hi and Glut1-Lo are shown. These percentages were used to generate the pie charts shown in Figure 6.
